# Exploring the glycolytic cross-talk genes between inflammatory bowel disease and colorectal cancer

**DOI:** 10.1007/s10142-023-01170-5

**Published:** 2023-07-10

**Authors:** Chenglin Ye, Yabing Huang, Yuan Gao, Sizhe Zhu, Jingping Yuan

**Affiliations:** 1 Department of Pathology, Renmin Hospital of Wuhan University, Wuhan, Hubei People’s Republic of China; 2 Department of Otolaryngology-Head and Neck Surgery, Tongji Hospital, Tongji Medical College, Huazhong University of Sciences and Technology, Wuhan, Hubei People’s Republic of China

**Keywords:** Inflammatory bowel disease, Colorectal cancer, Glycolysis, Cross-talk genes

## Abstract

Patients with inflammatory bowel disease (IBD) have a higher risk of developing colorectal cancer (CRC). Glycolysis is involved in the development of both IBD and CRC. However, the mechanisms and outcomes of glycolysis shared between IBD and CRC remain unclear. This study aimed to explore the glycolytic cross-talk genes between IBD and CRC integrating bioinformatics and machine learning. With WGCNA, LASSO, COX, and SVM-RFE algorithms, P4HA1 and PMM2 were identified as glycolytic cross-talk genes. The independent risk signature of P4HA1 and PMM2 was constructed to predict the overall survival rate of patients with CRC. The risk signature correlated with clinical characteristics, prognosis, tumor microenvironment, immune checkpoint, mutants, cancer stemness, and chemotherapeutic drug sensitivity. CRC patients with high risk have increased microsatellite instability, tumor mutation burden. The nomogram integrating risk score, tumor stage, and age showed high accuracy for predicting overall survival rate. In addition, the diagnostic model for IBD based on P4HA1 and PMM2 showed excellent accuracy. Finally, immunohistochemistry results showed that P4HA1 and PMM2 were significantly upregulated in IBD and CRC. Our study reveals the presence of glycolytic cross-talk genes P4HA1 and PMM2 between IBD and CRC. This may prove to be beneficial in advancing research on the mechanism of development of IBD-associated CRC.

**Supplementary Information:**

The online version contains supplementary material available at 10.1007/s10142-023-01170-5.

## Introduction

Inflammatory bowel disease (IBD) is one of the most prevalent diseases of the digestive system and includes two major subtypes, Crohn’s disease and ulcerative colitis (Hnatyszyn et al. 2019, Ng et al. 2017). Long-standing intestinal inflammation in patients with IBD disrupts the normal intestinal structure and leads to the dysregulation of the intestinal mucosal immune system (Hnatyszyn, Hryhorowicz, Kaczmarek-Rys, Lis, Slomski, Scott and Plawski 2019). Colorectal cancer (CRC) is the most common IBD-associated cancer. Patients with IBD have a 1.7-fold increased risk of developing CRC (Nadeem et al. 2020). CRC is also the most commonly diagnosed gastrointestinal cancer and ranks third in cancer-related deaths worldwide (Arnold et al. 2020, Biller and Schrag 2021, Kim et al. 2021). Although the incidence of CRC in China is lower than that in Western countries, China ranks first in the number of new cases of CRC and related deaths in the world owing to its comparatively large population (Yang et al. 2020). The association between IBD and CRC is unequivocal, but the mechanisms involved remain unclear.

Glycolysis is linked to the inflammatory response and immunoregulation. Glycolysis is involved in the development of several inflammation-related diseases, such as IBD (Riffelmacher et al. 2021), Alzheimer’s disease (Hipkiss 2019), SARS-CoV-2 infection (Duan et al. 2021), and rheumatoid arthritis (Abboud et al. 2018). Cancer development involves cell metabolic reprogramming, mainly via the Warburg effect, which is also termed aerobic glycolysis (Mao et al. 2022, Warburg 1956).

In this work, we aim to investigate the effect of glycolysis on IBD and CRC to reveal the cross-talk genes between IBD and CRC. Transcriptomic data from IBD and CRC patients retrieved from Gene Expression Omnibus (GEO) and The Cancer Genome Atlas (TCGA) were used to identify the glycolytic cross-talk genes. A network of co-regulated transcription factors of the cross-talk genes was constructed. We explored the relationship between the cross-talk genes and prognosis, tumor microenvironment (TME), mutations, and drug sensitivity in CRC. Moreover, a nomogram was constructed to predict the prognosis of CRC, and a combined diagnostic model of IBD was identified. Finally, the expression of cross-talk genes was validated by immunohistochemistry (IHC).

## Results

### Identification of differentially expressed glycolysis-related genes

**Fig. 1:**
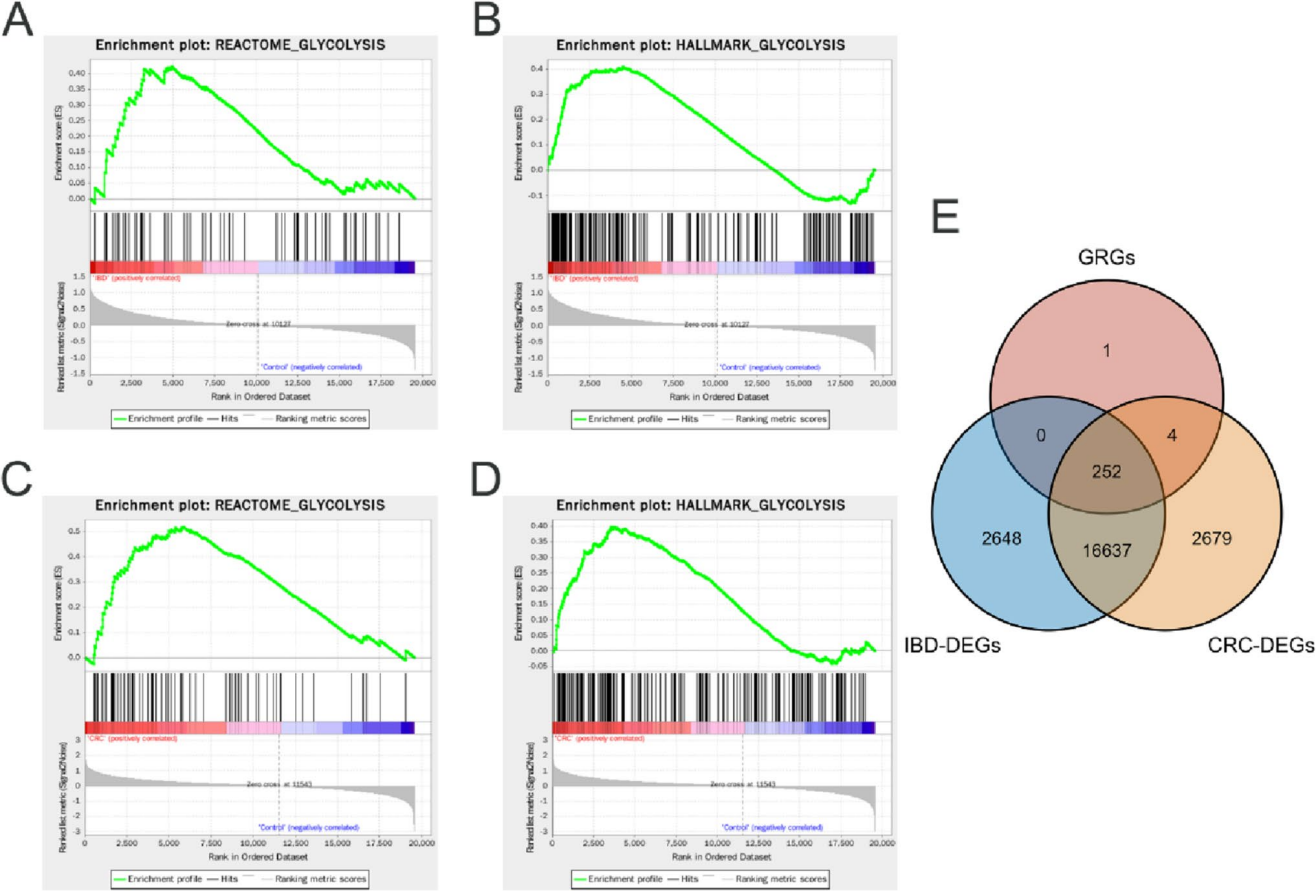
Glycolysis-related genes in IBD and CRC. **A**–**B** Enrichment plots of glycolysis in IBD using GSEA. **C**–**D** Enrichment plots of glycolysis in CRC using GSEA. **E** Intersection of GRGs, IBD-DEGs, and CRC-DEGs

The dataset containing 638 CRC and 49 normal samples with clinical information was obtained from TCGA-Colon Adenocarcinoma (COAD) Rectum Adenocarcinoma (READ). The IBD datasets containing 70 inflamed IBD tissues and 31 healthy controls were obtained from GSE17928. We downloaded glycolysis-related gene sets from MsigDB and GSEA was performed on the CRC and IBD datasets, respectively. As shown in Fig. 1A–D, HALLMARK_GLYCOLYSIS and REACTOME_GLYCOLYSIS were significantly enriched in CRC and IBD samples. The DEGs of IBD and CRC were identified via the R package limma. The overlap between IBD-DEGs, CRC-DEGs, and GRGs was defined as differential expression glycolysis-related genes (DE-GRGs) for further analysis (Fig. 1E).

### Weighted gene co-expression network analysis

**Fig. 2:**
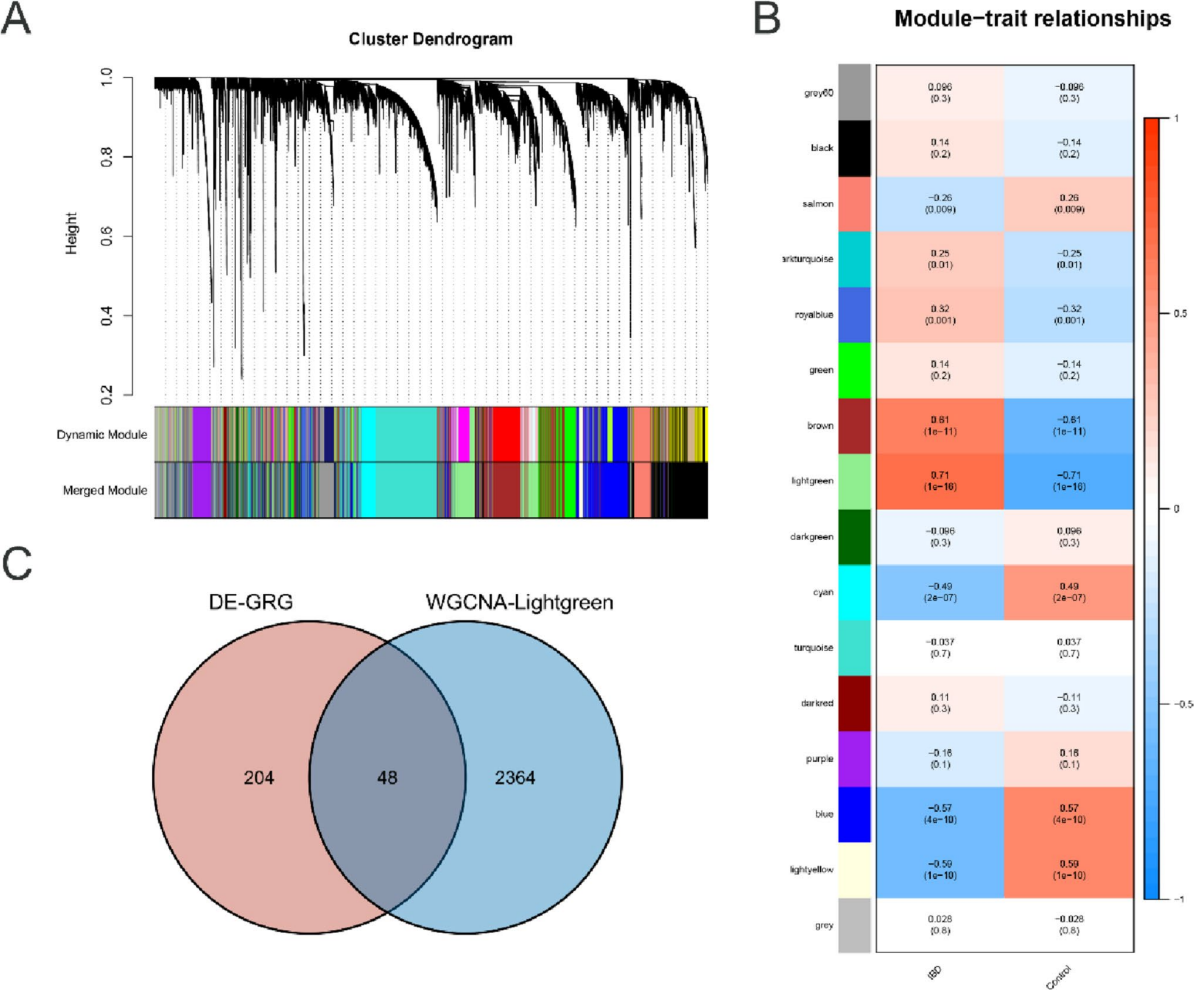
WGCNA. **A** Resulting gene dendrograms. **B** Module trait relationships in gene subtype A and B, which contained the corresponding correlation and *p* value. **C** Intersection of DE-GRG and lightgreen module

To explore the relative gene modules of IBD, WCGNA was applied in GSE17928 with a soft threshold at 6 (Figure S1A). Sixteen gene modules were identified, the lightgreen module in Fig. 2A–B, and Figure S1B show strong positive correlation with CRC, with module trait correlation = 0.71. The intersection of the lightgreen module and the DE-GRGs obtained included 48 genes (Fig. 2C).

### Screening for prognosis-related genes

**Fig. 3:**
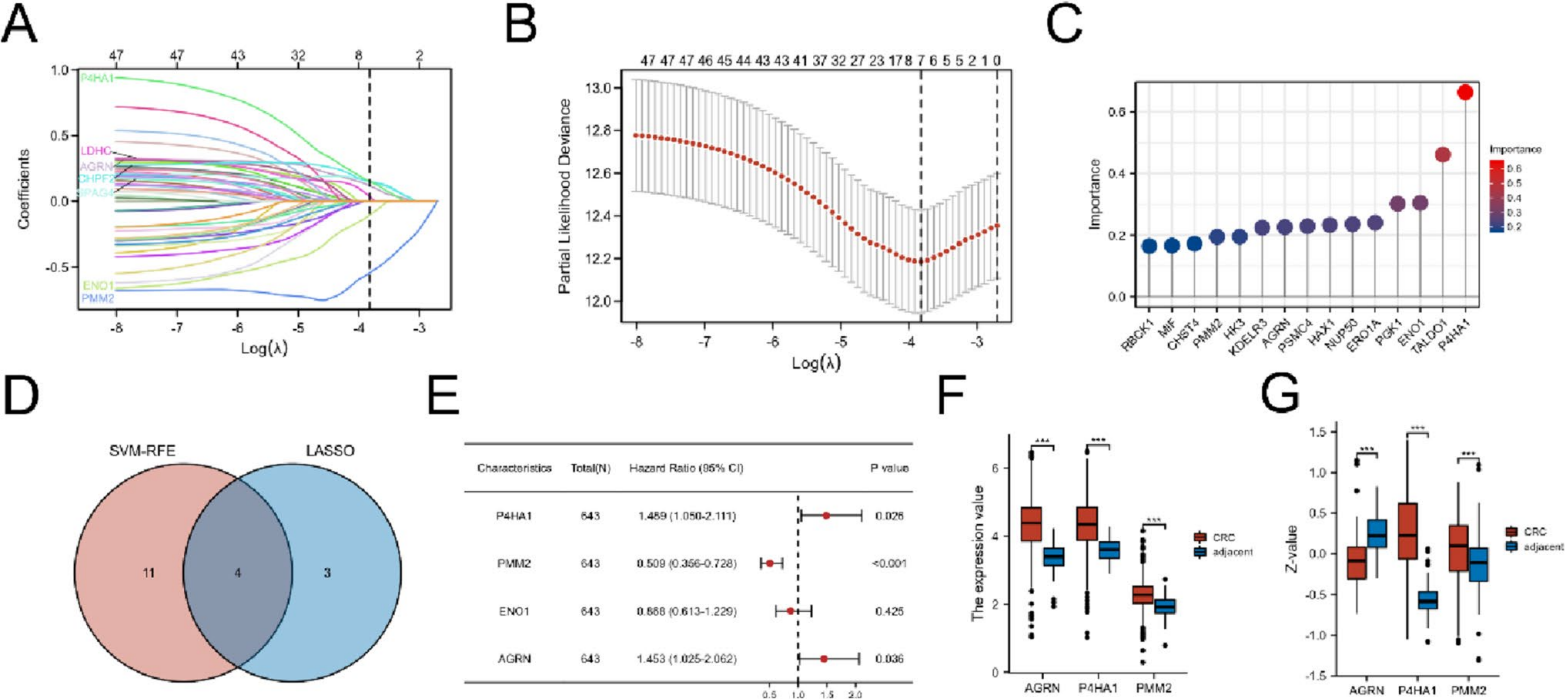
Screening prognostic genes in CRC. **A** Ten-fold cross-validation for the coefficients of hub genes in the LASSO model. **B** The selection of optimal parameter (lambda) in LASSO model. **C** Lollipop chart of importance in SVM-RFE. **D** Intersection of SVM-RFE and LASSO. **E** Forest plot of the multivariable Cox model. **F** Transcriptomic expression levels of AGRN, P4HA1, and PMM2. **G** Proteomic expression levels of AGRN, P4HA1, and PMM2. ***p < 0.001

**Fig. 4:**
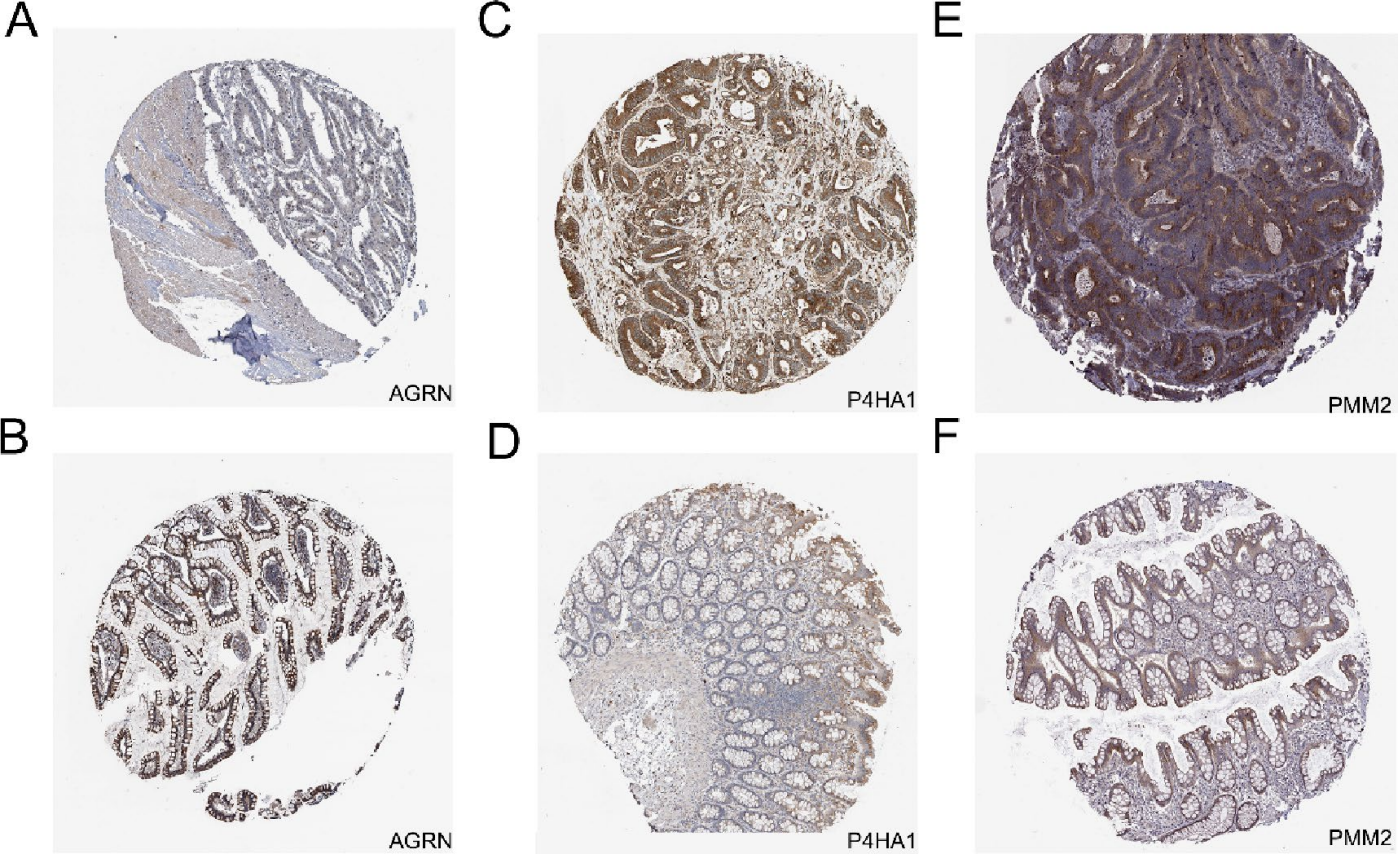
IHC validation in HPA database. **A**–**B** IHC of AGRN in normal and CRC, respectively. **C**–**D** IHC of P4HA1 in normal and CRC, respectively. **E**–**F** IHC of PMM2 in normal and CRC, respectively

The obtained 48 genes were used to explore prognostic markers in CRC via LASSO regression model and seven genes (P4HA1, PMM2, ENO1, LDHC, SPAG8, CHPF2, and AGRN) were obtained (Fig. 3A–B). SVM-RFE, a machine learning algorithm for feature selection, was applied to select prognosis-related genes ranking by parameter “Importance” (Fig. 3C). The intersection genes of LASSO and SVM-RFE were used for next analysis (Fig. 3D). We performed univariate Cox regression analysis. As shown in Fig. 3E, only P4HA1, PMM2, and AGRN exhibited significant correlation with overall survival (OS), and the transcriptomic expression of P4HA1, PMM2, and AGRN was significantly upregulated in tumor tissues (Fig. 3F). Furthermore, we retrieved proteomic data and IHC staining from the CPTAC and HPA databases to investigate proteomic expression levels of P4HA1, PMM2, and AGRN. As shown in Fig. 3G, P4HA1 and PMM2 were highly expressed, whereas the expression level of AGRN was low in CRC tissues. The proteomic expression levels of P4HA1, PMM2, and AGRN were further confirmed via IHC staining from the HPA database (Fig. 4A–F). Since proteomic expression levels of P4HA1 and PMM2 were consistent with transcriptomic expression levels, these two genes were identified as glycolytic cross-talk genes between IBD and CRC.

### Prediction of co-regulated transcription factors

**Fig. 5:**
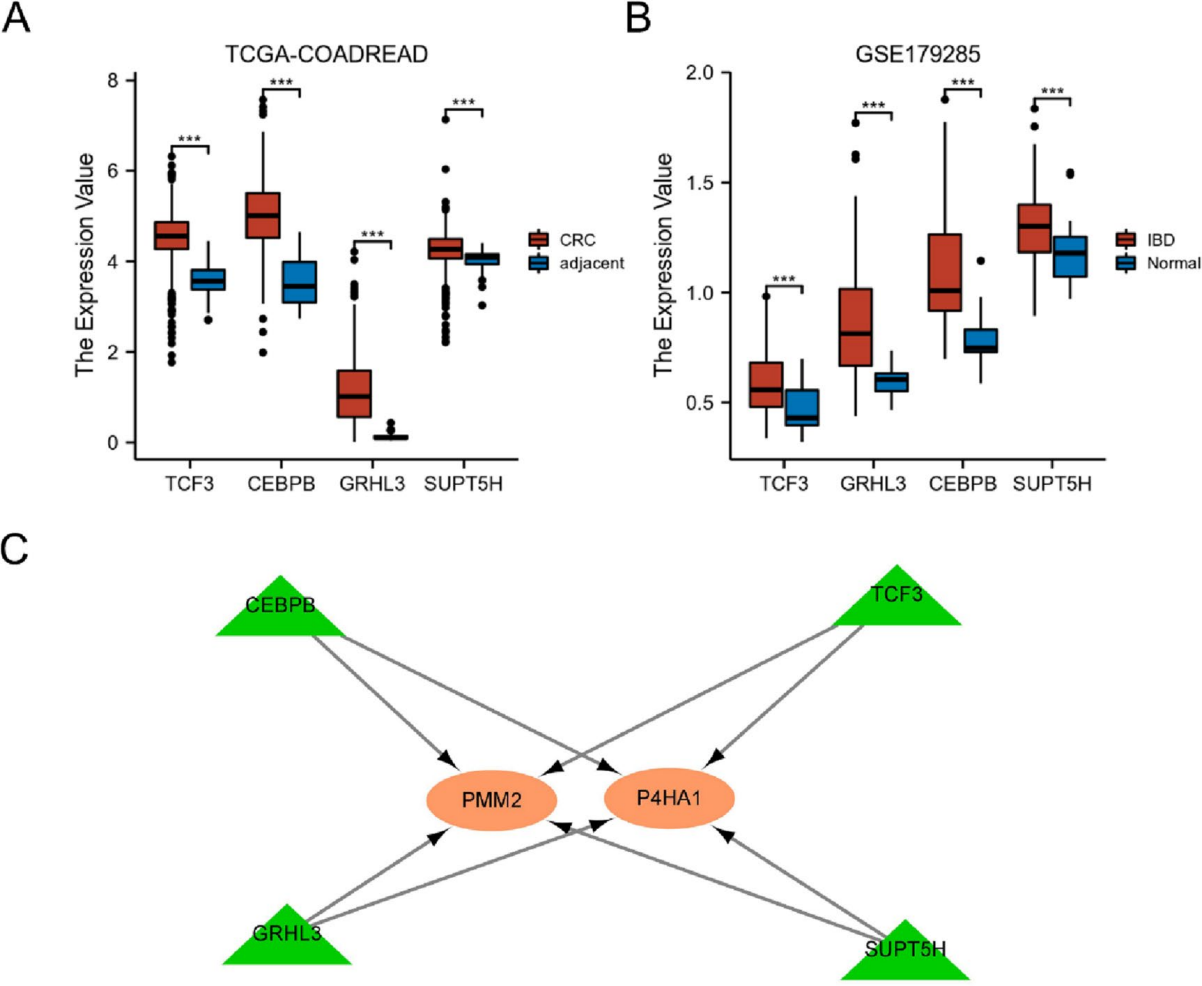
Co-related TF networks in CRC and IBD. **A** The expression of TCF3, CEBPB, GRHL3, and SUPT5H in CRC. **B** The expression of TCF3, CEBPB, GRHL3, and SUPT5H in IBD. **C** Co-related TF networks of P4HA1 and PMM2. ***p < 0.001

To explore the cross-talk of regulatory networks between P4HA1 and PMM2 in both IBD and CRC, the hTFtarget database was used to predict co-regulated TFs of P4HA1 and PMM2. One hundred twelve co-regulated TFs were found, and only four TFs (GRHL3, CEBPB, TCF3, and SUPT5H) were significantly upregulated in both IBD and CRC (Fig. 5A–B). Therefore, GRHL3, CEBPB, TCF3, and SUPT5H were used to construct the co-regulated network of P4HA1 and PMM2, which is presented in Fig. 5C.

### Construction of prognostic risk signature

**Fig. 6:**
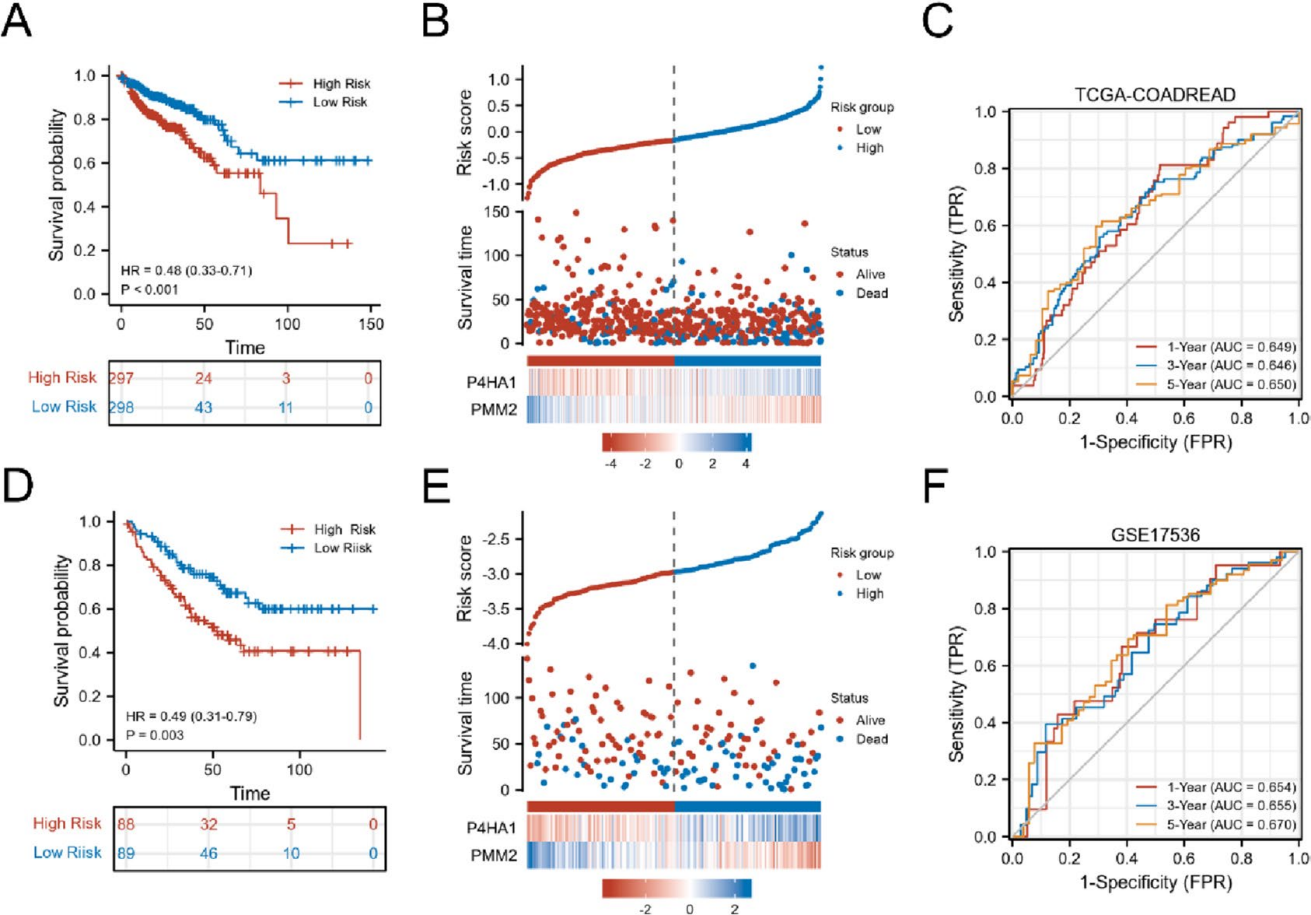
KM survival analysis, risk score assessment, and time-dependent ROC curves in the TCGA and GEO datasets. **A**–**C** TGCA-COADREAD. **D**–**F** GSE17536

To further explore the role of the two cross-talk genes, a prognostic signature was established as follows: Risk score = (0.3419)*P4HA1 + (−0.7116)*PMM2. Risk scores of each patient in the TCGA-COADREAD and GSE17536 datasets were calculated, and the patients were divided into a high risk group and a low risk group based on the median risk score. As shown in Fig. 6A, high risk patients had relatively low OS than patients with low risk in TCGA-COADREAD (*p* = 0.00015). The risk scores, survival status, and heatmap of P4HA1 and PMM2 are presented in Fig. 6B. The AUC values of TCGA-COADREAD at 1, 3, and 5 years were 0.649, 0.646, and 0.650, respectively (Fig. 6C). The prognostic signature of the two genes was validated in the GSE17536 dataset. Similarly, in the TCGA-COADREAD dataset, patients with low risk had relatively higher OS than patients in the high risk group (Fig. 6D). The risk scores, survival status, and heatmap of P4HA1 and PMM2 are presented in Fig. 6E. The AUC values of GSE17536 at 1, 3, and 5 years were 0.654, 0.655, and 0.670, respectively (Fig. 6F).

### Clinical correlation analysis

**Fig. 7:**
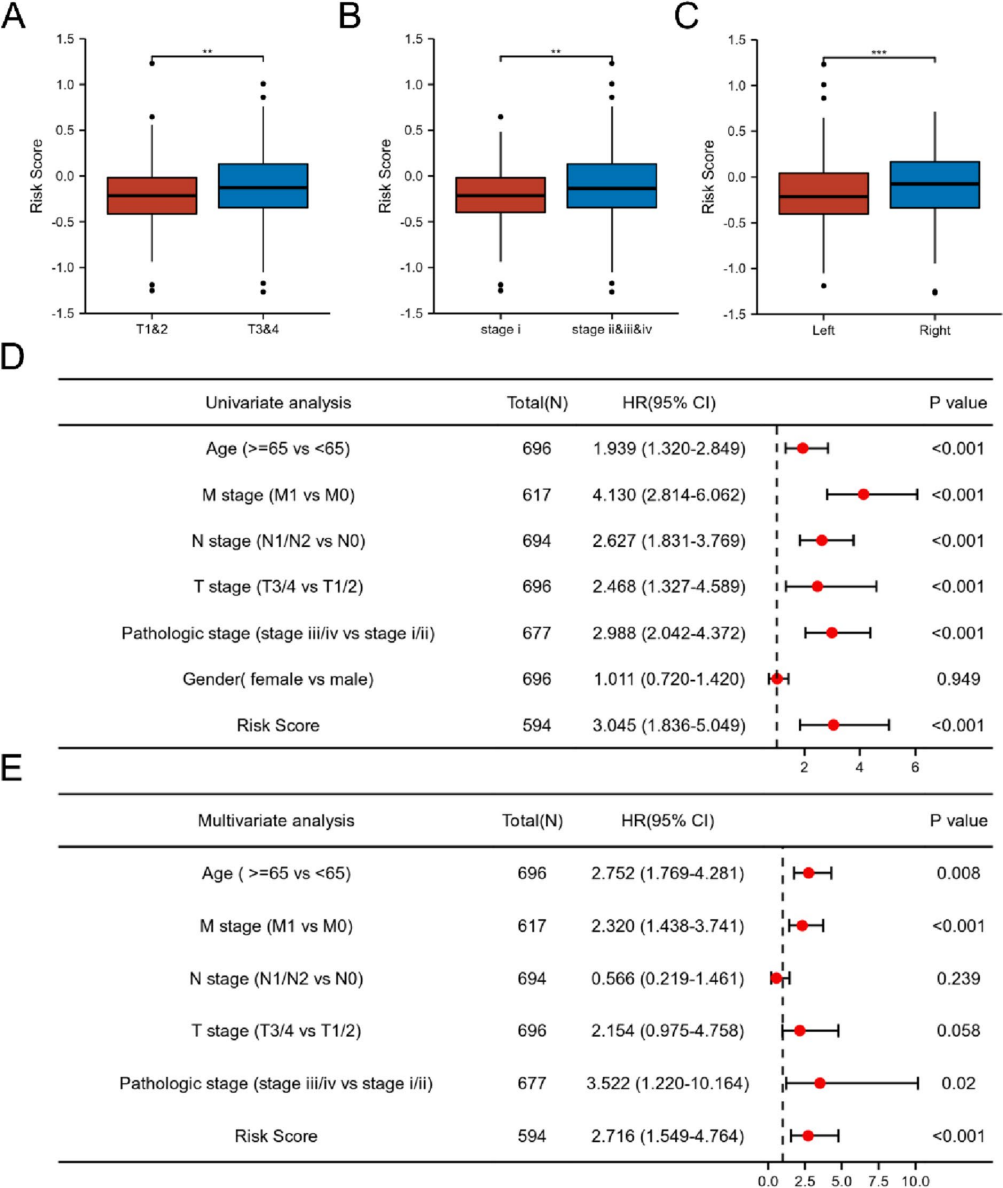
The relationship between risk score and clinical feature. **A** Boxplot of risk score in T stages. **B** Boxplot of risk score in pathological stage. **C** Boxplot of risk score in tumor location. **D** Univariate analysis of risk score. **E** Multivariate analysis of risk score. **p < 0.01, ***p < 0.001

We conducted correlation analysis between clinical characteristics and risk scores to explore the impact of risk scores on clinical characteristics. As shown in Fig. 7A, risk scores of patients in T stage 3 and 4 were significantly higher than those in T stage 1 and 2. In addition, we observed significantly higher risk scores for patients in stage II/III/IV relative to patients in stage I (Fig. 7B). Moreover, risk scores in right-side CRC were higher than that in left-side CRC (Fig. 7C). However, risk scores were not correlated with age and gender (Figure S2A–B). Univariate and multivariate Cox regression analyses were performed to estimate prognostic potential of the signatures of the two genes with several clinic pathological characteristics, such as age, pathological stage, T stage, M stage, N stage, and gender in CRC. As shown in Fig. 7D, the risk scores significantly correlated with OS (HR = 3.045, 95% CI = 1.836–5.049, *p* < 0.001) in univariate Cox regression. After correcting for other confounding factors, the risk score (HR = 2.716, 95% CI = 1.549–4.764, *p* < 0.001), age ≥ 65 (HR = 2.752, 95% CI = 1.769–4.281, *p* = 0.008), and M stage (HR = 2.320, 95% CI = 1.438–3.741, *p* < 0.001) still proved to be independent predictors for OS in the multivariate Cox regression analysis (Fig. 7E). These results demonstrate that risk scores could be a potential prognostic indicator for predicting OS in CRC.

### Characteristics of tumor microenvironment and immune checkpoints

**Fig. 8:**
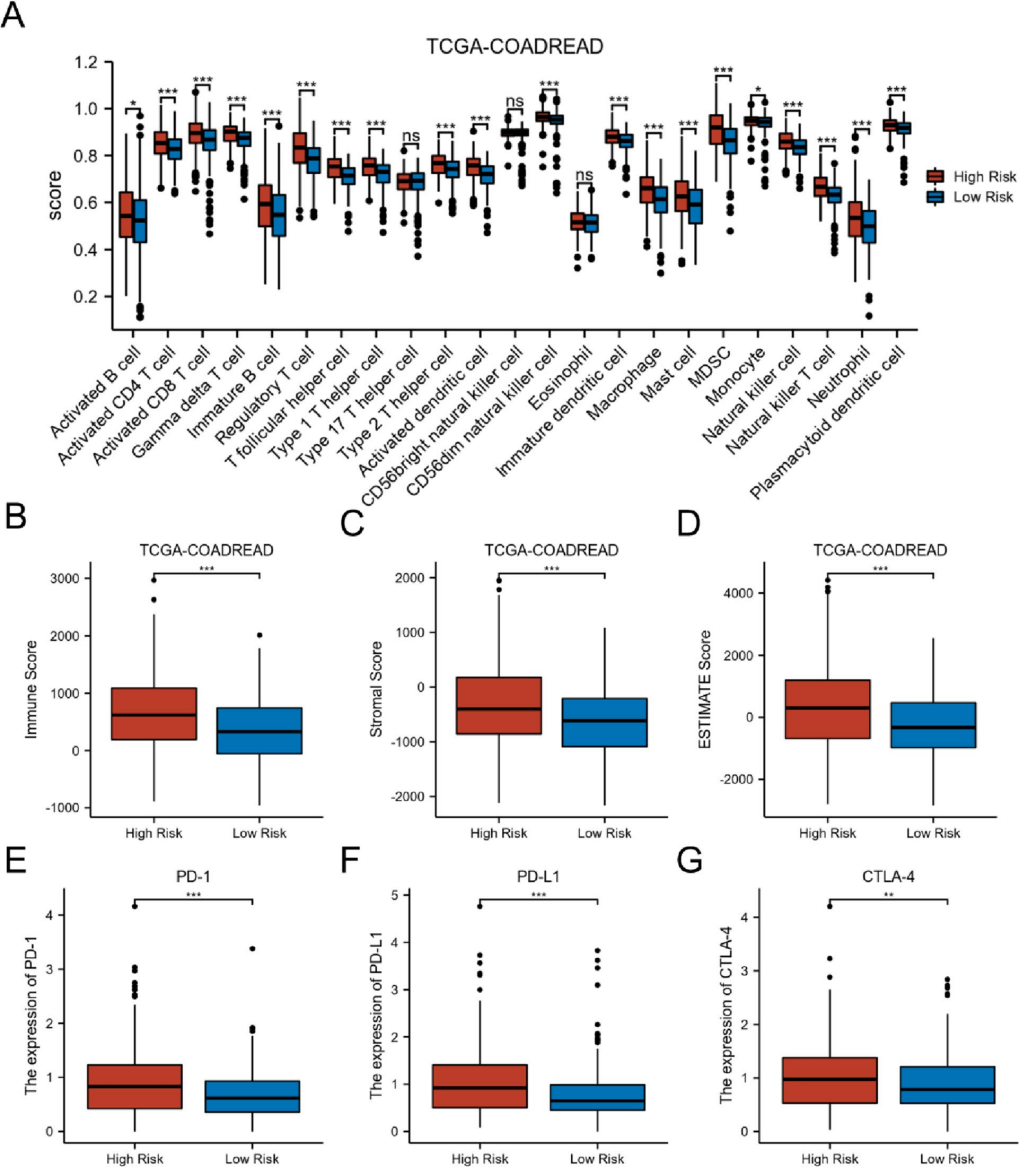
Immune characteristics between high and low risk group in TCGA dataset. **A** Infiltration of 23 immune cells using ssGSEA. **B**–**D** Immune, stromal, and ESTIMATE scores. **E**–**G** The expression of PD-1, PD-L1, and CTLA-4. *p < 0.05, **p < 0.01, ***p < 0.001, ns not significant

ssGSEA was used to estimate immune cell infiltration in CRC patients from TCGA-COADREAD and GSE17536 datasets. As shown in Fig. 8A and Figure S3A, higher infiltration levels were observed in the high risk group relative to those in the low risk group in both TCGA-COADREAD and GSE17536. To explore tumor microenvironment (TME) of CRC, ESTIMATE was applied to calculate the immune score, stromal score, and ESTIMATE score in TCGA-COADREAD and GSE17536. The immune score, stromal score, and ESTIMATE scores were significantly higher in the high risk group than in the low risk group (Fig. 8B–D and Figure S3B–D). Considering the vital role of immune checkpoint molecules (PD-1, PD-L1, and CTLA-4) in the tumor immune microenvironment, we analyzed the expression of PD-1, PD-L1, and CTLA-4 in the two groups. As shown in Fig. 8E–G, the expression of all immune checkpoint molecules was significantly elevated in the high risk group.

### Correlation of gene signature with microsatellite instability and stemness

**Fig. 9:**
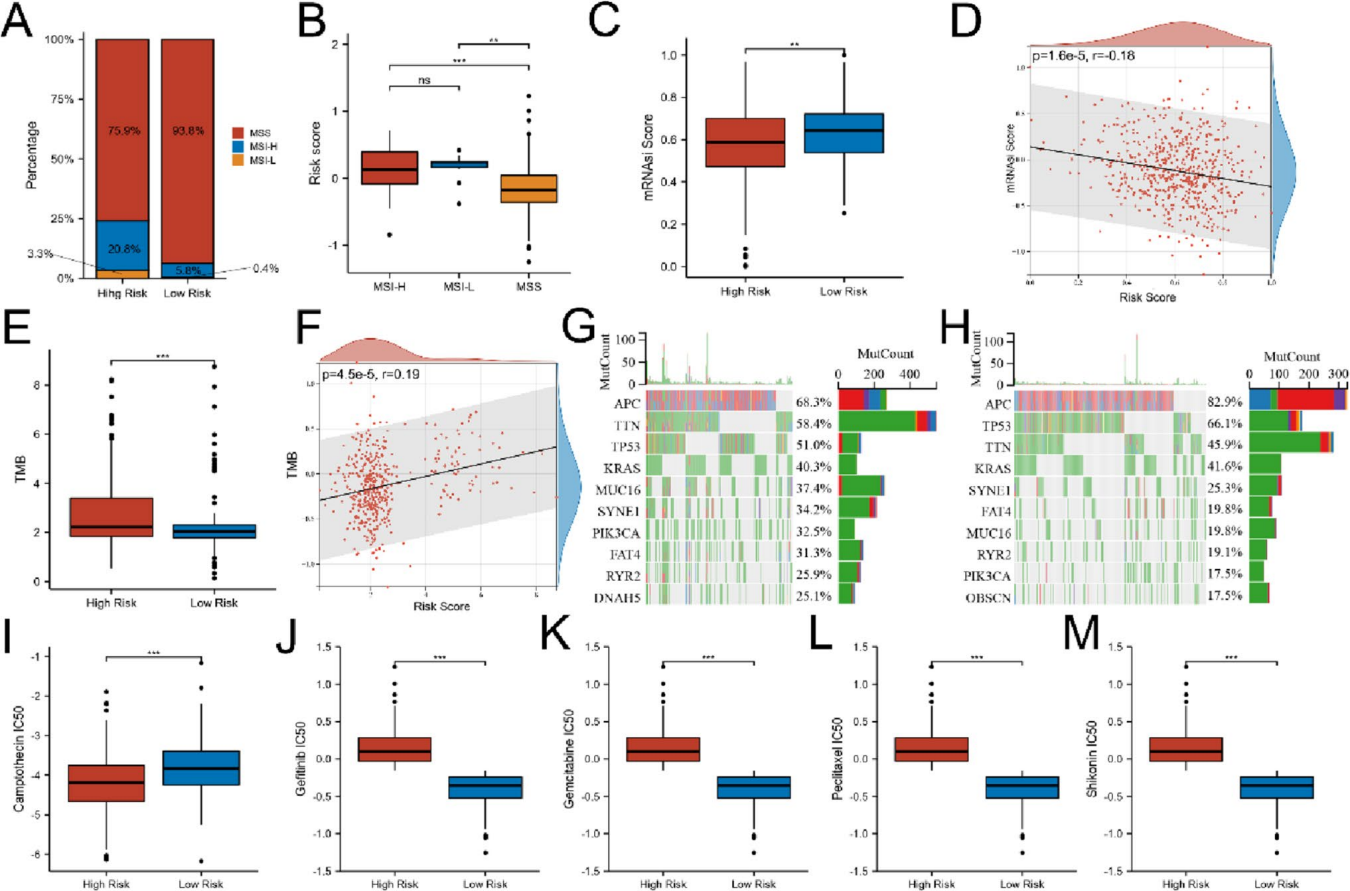
Mutant and drug susceptibility analysis. **A**–**B** Relationship between MSI and risk score. **C**–**D** Relationship between mRNAsi score and risk score. **E**–**F** Relationship between TMB and risk score. **G**–**H** The waterfall plot of somatic mutation characteristics of CRC patients with high and low risk. **I**–**M** Relationships between chemotherapeutic sensitivity and risk score. **p < 0.01, ***p < 0.001, ns not significant

A recent study reported that patients with high microsatellite instability (MSI-H) were more sensitive to immunotherapy (Ganesh et al. 2019). The MSI and microsatellite stable (MSS) status of CRC patients were estimated in the TGCA-CORADREAD dataset. As shown in Fig. 9A–B, high risk patients were significantly associated with MSI-H, whereas low risk patients were associated with MSS. Furthermore, we evaluated cancer stemness by mRNAsi. Correlation analysis revealed that risk scores were negatively associated with mRNAsi, indicating that CRC cells with a lower risk score had more distinct stem cell properties and a lower degree of cell differentiation (Fig. 9C–D).

### Mutation and drug sensitivity analysis

Increasing evidence suggests that patients with high tumor mutational burden (TMB) may benefit from immunotherapy because they have higher numbers of neoantigens (Snyder et al. 2014). Therefore, TMB scores were calculated in CRC. The CRC patients with high risk scores had higher TMB scores (Fig. 9E–F). We evaluated the variations in the distribution of the somatic mutations in the TCGA-COADREAD dataset. As shown in Fig. 9G, the ten most frequently mutated genes in the high risk group were APC, TTN, TP53, KRAS, MUC16, SYNE1, PIK3CA, FAT4, RYR2, and DHAN5. However, patients with a low risk had markedly higher frequencies of APC, TP53, and KRAS mutations compared to patients with a high risk (Fig. 9H). Furthermore, we investigated sensitivities of CRC patients to the commonly used chemotherapy drugs. We found that patients with a high risk score had a lower IC50 value for camptothecin, whereas IC50 values of gemcitabine, paclitaxel, gefitinib, and shikonin were significantly higher in high risk group (Fig. 9I–M).

### Establishment of a nomogram

**Fig. 10:**
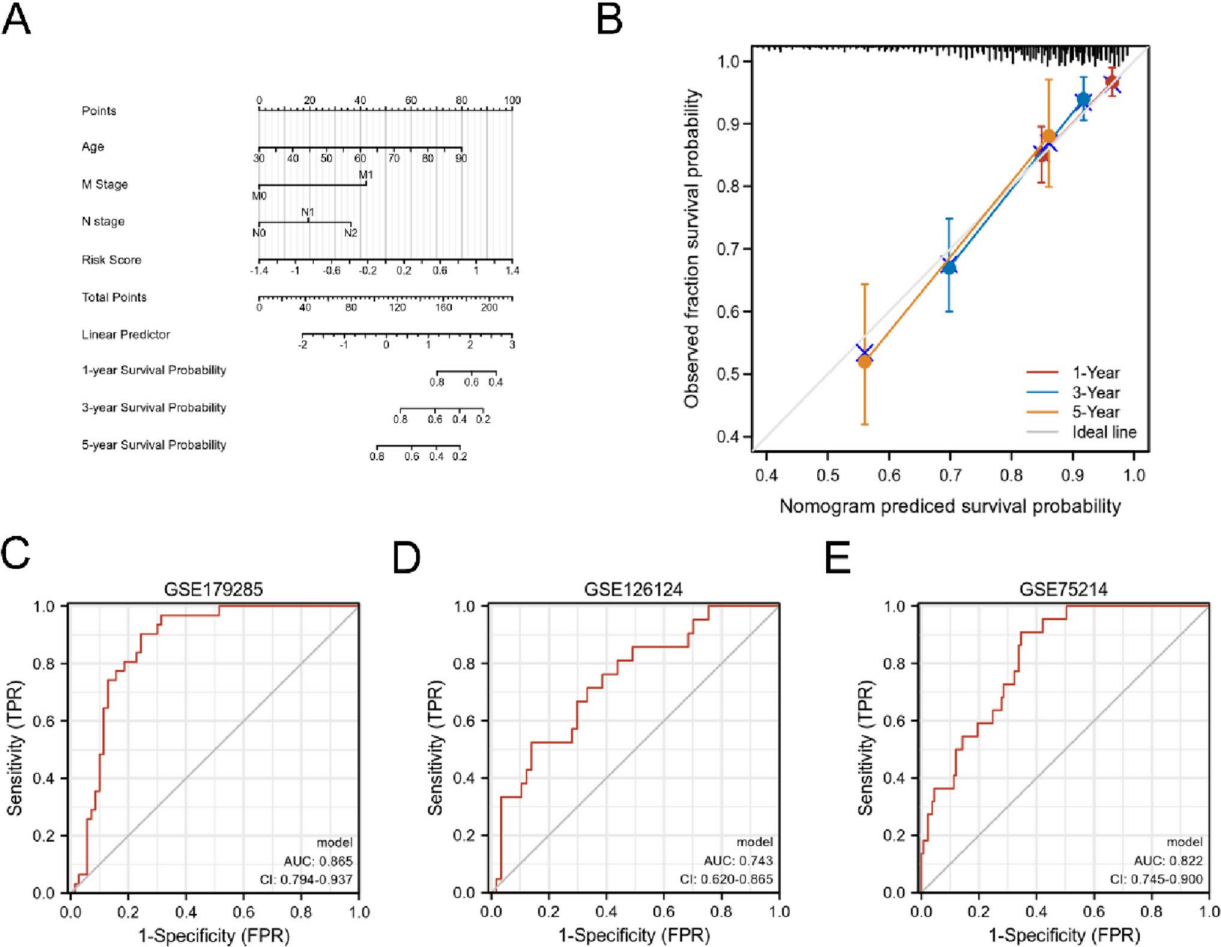
Predict and diagnostic values of signature in CRC and IBD. **A** A nomogram for predicting 1-, 3-, and 5-year OS of CRC patients. **B** Calibration curves of the nomogram for predicting 1-, 3-, and 5-year OS of CRC patients. **C**–**E** ROC of logistics model of P4HA1 and PMM2 in GSE179285, GSE12624, and GSE75214

To establish a quantitative prediction model, a nomogram was constructed (Fig. 10A), including risk score, age, M stage, and N stage. Calibration plots indicated that the nomogram and ideal model showed high consistency in TCGA-COADREAD (Fig. 10B).

### Diagnostic values of P4HA1 and PMM2 in IBD

We combined P4HA1 and PMM2 via a logistics model for ROC analysis. The results were presented in Fig. 10C–E. The AUC values of the combined model were 0.865, 0.743, and 0.822 in GSE179285, GSE126124, and GSE75214, respectively. This indicates that a combined model of P4HA1 and PMM2 performs well for diagnosing IBD.

### Validation of P4H1A and PMM2 expression via IHC

**Fig. 11:**
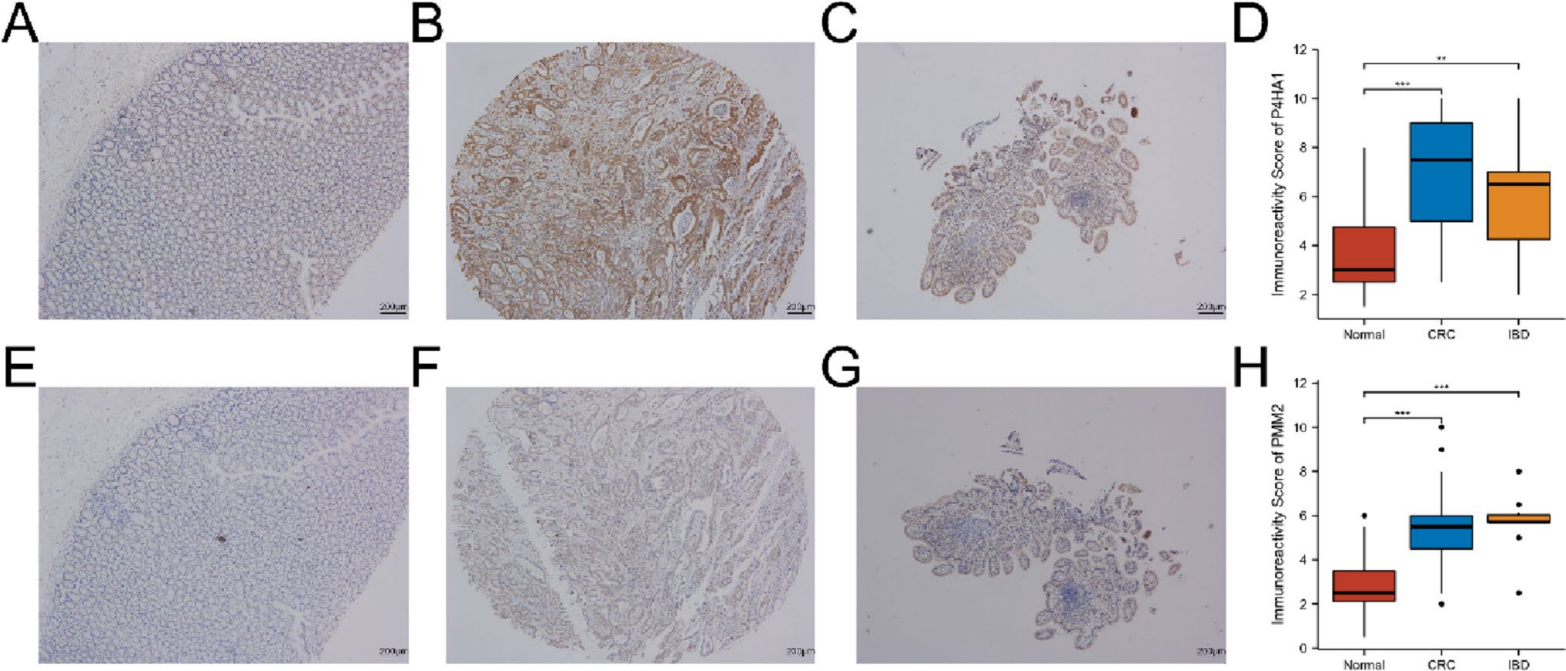
IHC validation (magnification ×40). **A**–**C** IHC of P4HA in normal, CRC, and IBD, respectively. **D** Immunoreactivity score of P4HA1 in normal, CRC, and IBD, respectively. **E**–**G** IHC of PMM2 in normal, CRC, and IBD, respectively. **H** Immunoreactivity score of PMM2 in normal, CRC, and IBD, respectively. **p < 0.01, ***p < 0.001

To validate the proteomic expression levels of P4HA1 and PMM2 in patients with IBD and CRC, we collected twenty IBD, one hundred CRC, and fifty normal formalin-fixed, paraffin-embedded tissue samples from the Renmin Hospital of Wuhan University and performed IHC. As shown in Fig. 11A–H, the proteomic expression levels of P4HA1 and PMM2 were significantly upregulated in IBD and CRC relative to normal tissues. Our results suggest that the glycolysis-related genes P4HA1 and PMM2 are involved in the pathogenesis of IBD and CRC.

## Discussion

Accumulating evidence suggests that patients with IBD have an increased risk of CRC (Beni et al. 2022, Rajamaki et al. 2021, Wijnands et al. 2021). It may be due to the development of dysplastic lesions in the colonic mucosa caused by chronic inflammation (Frigerio et al. 2021). Though the incidence of IBD-associated CRC is low relative to sporadic CRC, patients with IBD have a mortality rate of 10 to 15% due to CRC (Eluri et al. 2017). Glycolysis is one of the major metabolic pathways essential for providing energy for cellular processes, and it is involved in the regulation of innate and adaptive immune cells in the inflammatory response (Soto-Heredero et al. 2020). Studies have shown that macrophages regulate glycolysis via the B-cell adapter for PI3K to improve experimental colitis (Irizarry-Caro et al. 2020). Moreover, N6-methyladenosine modifications of HK2 and GLUT1 regulate glycolysis to enhance colorectal cancer progression (Shen et al. 2020).

IBD and CRC may have overlapping pathogenic pathways involving glycolysis. The main purpose of this study was to reveal glycolytic cross-talk genes in IBD and CRC. We firstly identified 252 cross-talk DE-GRGs between IBD and CRC. Further, WGCNA was used to screen for IBD-related modules, and the module with high trait correlation was intersected with DE-GRGs for the next step in the analysis. Finally, P4HA1 and PMM2 were identified as potential prognostic markers in CRC using LASSO, SVM-RFE, and Cox regressions. Therefore, we speculated that P4HA1 and PMM2 might be two glycolytic cross-talk genes. By further verification, we found that four TFs that participate in a coordinated manner in the regulation of P4HA1 and PMM2 were highly expressed in IBD and CRC, including GRHL3, CEBPB, TCF3, and SUPT5H.

One of two glycolytic cross-talk genes identified in this study, P4HA1, is a key enzyme in collagen synthesis. Upregulation of P4HA1 contributes to glycolysis by improving the stability of HIF1-α in breast cancer and pancreatic cancer (Cao et al. 2019, Xiong et al. 2018). Studies have confirmed P4HA1 is overexpressed in CRC. Agarwal et al. reported that knockdown of P4HA1 decreased CRC cell proliferation, invasion, and migration and inhibited tumor growth as well as metastases. P4HA1 inhibitors have been shown to be an effective therapeutic strategy for aggressive CRC (Agarwal et al. 2020). The other glycolytic cross-talk gene identified was PMM2. PMM2 catalyzes the isomerization of mannose 6-phosphate to mannose 1-phosphate, which is a precursor of GDP-mannose necessary for the synthesis of dolichol-P-oligosaccharides. Mutations of PMM2 result in the PMM2-congenital disorder of glycosylation, which is an inherited condition that affects many parts of the body (Witters et al. 2019). Studies have indicated that the overexpression of PMM2 could promote malignancy of renal cell carcinoma (Yamada et al. 2018).

To further explore the role of P4HA1 and PMM2, we constructed a risk signature based on P4HA1 and PMM2 in CRC. Our results suggest that CRC patients with a high risk score have a poor prognosis. Moreover, patients with low and high risk scores showed significantly different clinical and pathological characteristics, mutations, TMEs, immune checkpoints, MSI, cancer stemness, and drug susceptibility. Finally, a nomogram was constructed by integrating the risk score, tumor stage, and age which further facilitated the use of risk signature. These findings may improve the prognosis stratification of patients with CRC and provide new ideas for targeted therapies. Despite recent advances in immunotherapy for CRC, different patients still show different sensitivity to treatment, highlighting the crucial role of TME in CRC tumorigenesis and progression (Chen et al. 2021). PD-1/PD-L1 and CTLA-4 blockades have demonstrated their safety and efficacy and have been used to treat CRC (Monjazeb et al. 2021, Yaghoubi et al. 2019). Our results showed that patients with a high risk score had higher infiltration of immune cells and high expression levels of immune checkpoints. Moreover, TMB score and the proportion of patients with MSI-H were higher in the high risk group. Previous studies have shown that patients with a high TMB or MSI-H may be more sensitive to immunotherapy (Ganesh, Stadler, Cercek, Mendelsohn, Shia, Segal and Diaz 2019, Snyder, Makarov, Merghoub, Yuan, Zaretsky, Desrichard, Walsh, Postow, Wong, Ho, Hollmann, Bruggeman, Kannan, Li, Elipenahli, Liu, Harbison, Wang, Ribas, Wolchok and Chan 2014). This suggests that CRC patients with a high risk score are more likely to benefit from immunotherapy. Furthermore, we evaluated the diagnostic values of P4HA1 and PMM2 in IBD. Our results reveal that a combined model based on P4HA and PMM2 had an excellent diagnostic accuracy for IBD.

Nevertheless, this study had several limitations. Firstly, although we applied IHC to validate the expression levels of P4HA1 and PMM2 in patients with IBD and CRC, it lacks some clinical information, such as disease activity, previous therapies, and prognosis, which may have affected expression levels. Secondly, we failed to find an expression profile data of IBD-associated CRC to further validate our findings.

## Conclusion

Our comprehensive analyses revealed glycolysis may be involved in IBD developing CRC. Furthermore, glycolysis-related genes P4HA1 and PMM2 are potential cross-talk genes and biomarkers in IBD and CRC. This study provides new insights for the further study of the molecular mechanism of IBD-associated CRC.

## Method

### Sample collection and immunohistochemistry analysis

Formalin-fixed, paraffin-embedded tissue samples were collected from twenty IBD, one hundred CRC, and fifty healthy patients for IHC analysis from the Renmin Hospital of Wuhan University from 2020 to 2021. This study was approved by the Ethics Committee of Renmin Hospital of Wuhan University (WDRY2021-KS066); all methods were performed in accordance with the relevant guidelines and the Declaration of Helsinki. Immunohistochemical staining was performed using a standard EnVision complex method (Kammerer et al. 2001). The primary antibodies used were P4HA1 (Proteintech, Wuhan, China) and PMM2 (Proteintech, Wuhan, China). Five high-power fields (400×) were randomly selected, and examination and scoring were performed independently by three experienced pathologists who were unaware of the clinical information. The expression of P4HA1 and PMM2 was calculated by multiplying the mean signal intensity (on a scale of 0–3: 0, no staining; 1, light staining; 2, moderate staining; and 3, strong staining) and the proportion of positive tumor cells (on a scale of 0–4: 0, 0%; 1, 1–25%; 2, 26–50%; 3, 51–75%; and 4, 76–100%). The final immunoreactive scores were the mean of scores from the three pathologists.

### Data collection and gene set enrichment analysis

The RNA sequencing data and clinical data of 644 CRC patients were downloaded from TCGA. The expression array profiles of CRC patients were obtained from GEO, namely, GSE161158 (*n* = 192) and GSE17536 (*n* = 178). The IBD datasets GSE179285 (70 inflamed IBD tissues and 31 healthy controls), GSE75214 (133 inflamed IBD tissues and 22 healthy controls), and GSE126124 (57 inflamed IBD tissues and 21 healthy controls) were used. Gene set enrichment analysis (GSEA) was performed via GSEA/MSigDB and visualized by the R package ggplot2 (Subramanian et al. 2005, Wickham 2009). Glycolysis-related pathways from GSEA were used to obtain glycolysis-related genes (GRGs).

### Differential expression and weighted correlation network analysis

Differential expression analyses were performed using the R package limma with a false discovery rate of less than 0.05 (Ritchie et al. 2015), then genes were defined as IBD-differential expression genes (DEGs) and CRC-DEGs. Weighted correlation network analysis (WGCNA) was applied to assess the relative importance and module membership of genes (Langfelder and Horvath 2008). The minimum number of module genes was set at 30. The hierarchical clustering dendrogram summarizes the gene modules with different colors.

### Construction and evaluation of the prognostic model

The least absolute shrinkage and selection operator (LASSO) regression and support vector machine recursive feature elimination (SVM-RFE) were applied to screen CRC prognosis and to construct a prognosis-predicting model using the glmne R package (Friedman et al. 2010). The risk score was calculated using the following formula: Risk score = ${\sum}_{i=1}^n coef\ast id$ (Chen et al. 2007). The survminer and survival R packages were used to conduct the Kaplan–Meier survival analysis to assess the difference in survival between high and low risk score groups. We estimated the performance of the gene risk model using the time-dependent receiver operating characteristic (ROC) curve estimated by the R package survivalROC (Lorent et al. 2014). Then, Cox regression analyses were conducted to estimate the independent prognostic values of signature and other clinical characteristics. The proteomic expression of prognostic cross-talk genes was confirmed by the Clinical Proteomic Tumor Analysis Consortium (CPTAC) and the Human Protein Atlas (HPA) databases (Uhlen et al. 2015). To estimate survival likelihood, a nomogram was constructed based on the risk score and clinical characteristics using the R package rms, and the prognostic ability of the nomogram was assessed using calibration plots.

### Prediction of co-regulated transcription factors

The hTFtarget database was utilized to predict co-regulated transcription factors (TFs) (Zhang et al. 2020). hTFtarget is an online tool that has a curated and comprehensive database of TF-target regulators from large-scale ChIP-Seq data from human TFs (7190 experimental samples of 659 TFs), in 569 conditions (399 types of cell line, 129 classes of tissues or cells, and 141 kinds of treatments). The co-regulation network was constructed and visualized via Cytoscape.

### Evaluation of immune cell infiltration and tumor microenvironment

Single-sample GSEA (ssGSEA) was applied to estimate the 23 common immune cell infiltrations (Charoentong et al. 2017). The R package estimate was used to predict the presence of stromal or immune cells in tumor tissues (Yoshihara et al. 2013). The one-class logistic regression algorithm constructed by Malta et al. was used to evaluate cancer stemness based on the mRNA expression-based stemness index (mRNAsi) (Malta et al. 2018).

### Mutations and drug susceptibility analysis

Files in the mutation annotation format from the TCGA database were generated by the R package maftools for mutation analysis and the calculation of tumor mutation burden (TMB) score (Mayakonda et al. 2018). The semi-inhibitory concentration (IC50) values of chemotherapeutic drugs commonly used to treat CRC were obtained using the R package pRRophetic (Geeleher et al. 2014).

## Supplementary information

ESM 1 (DOCX 1053 kb)

## Data Availability

The data used to support the findings of this study are available from supplementary materials and the corresponding author upon request.
