# Supplementary material for: Exploring the glycolytic cross-talk genes between inflammatory bowel disease and colorectal cancer
Source: Funct Integr Genomics. 2023 Jul 10;23(3):230. doi: 10.1007/s10142-023-01170-5 (PMC10333365; doi:10.1007/s10142-023-01170-5)
Supplement: Supplementary file 1 — (DOCX 1053 kb) [file 10142_2023_1170_MOESM1_ESM.docx]

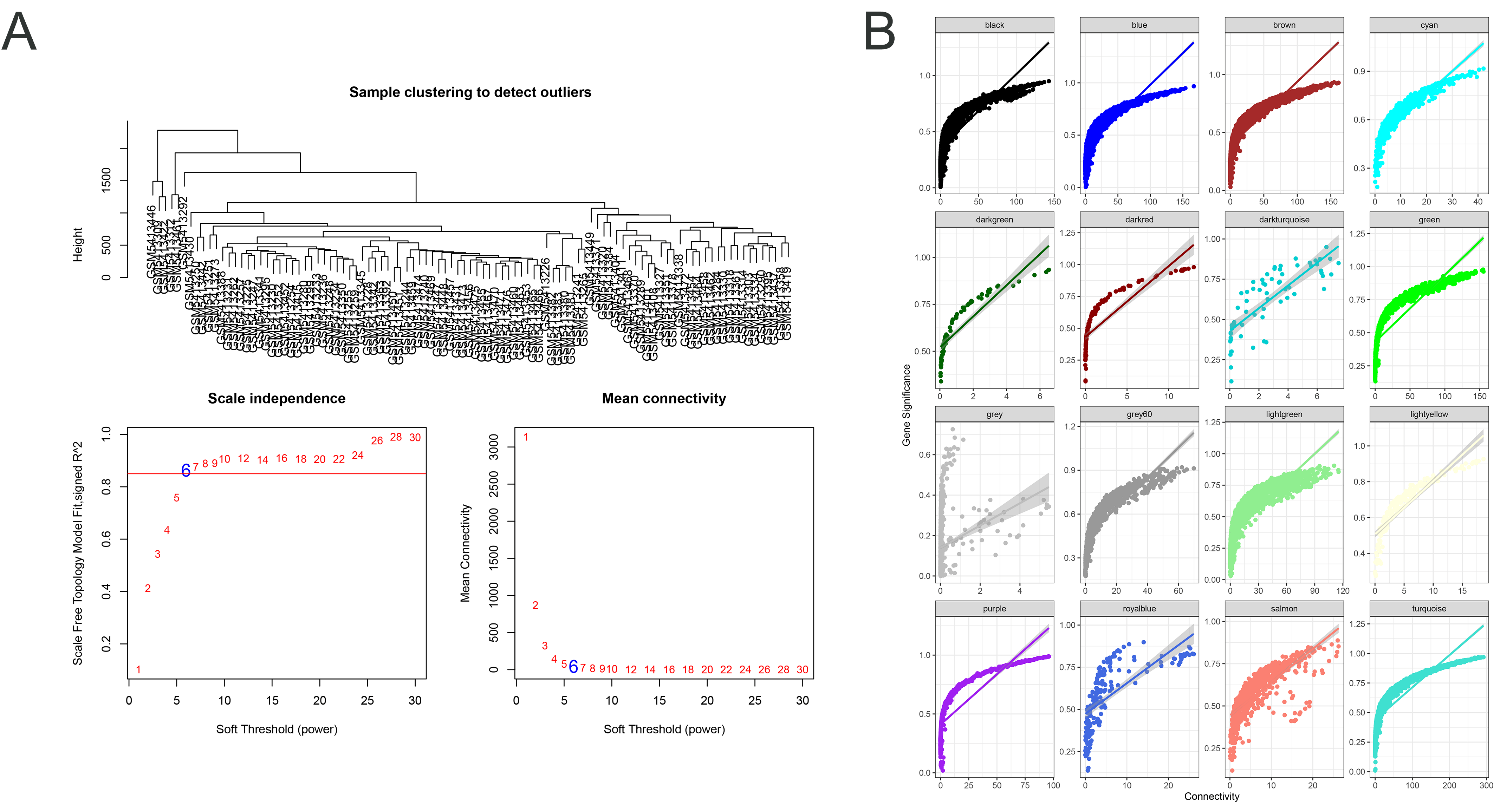


**Supplementary Figure S1.** (A) WGCNA clustering and soft threshold β setting. (CDF) curves. (B) Relationship of connectivity and gene significance.


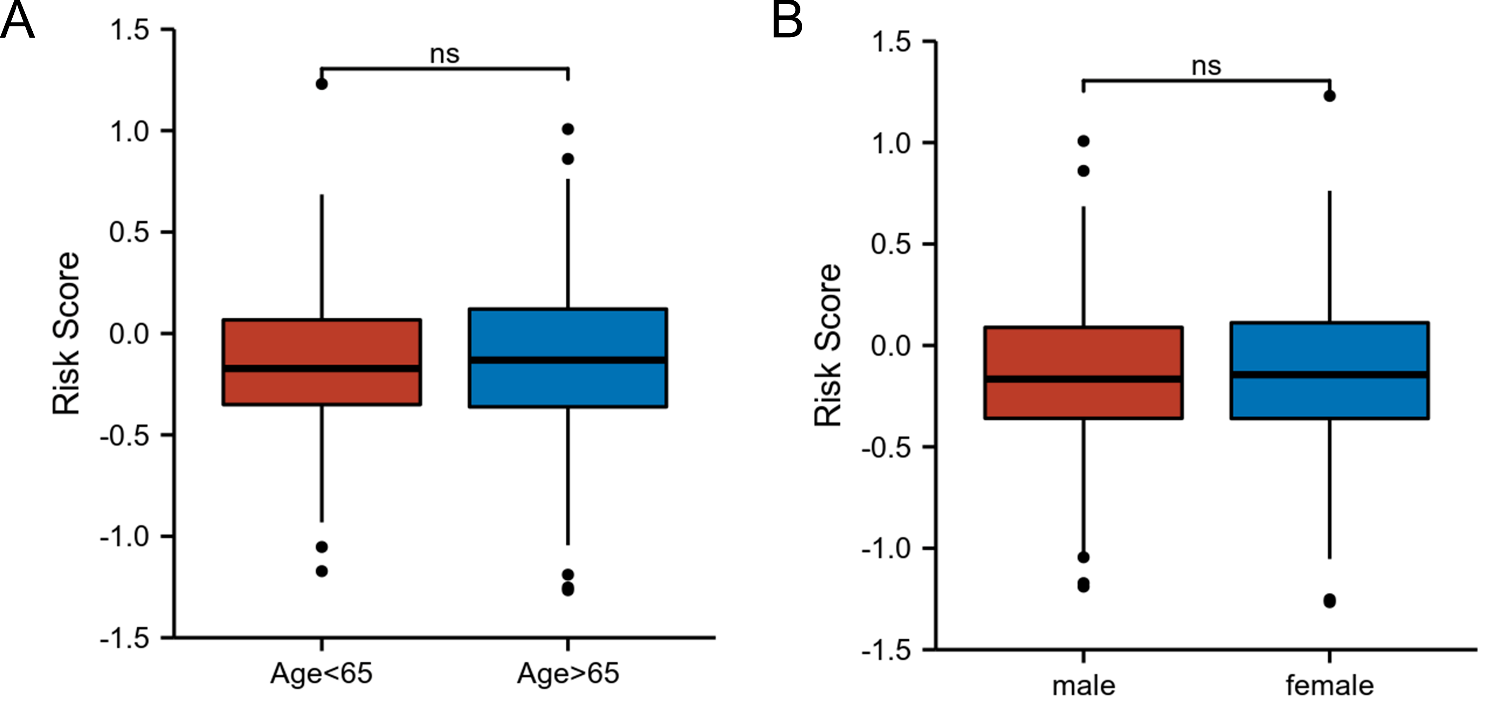


**Supplementary Figure S2.** (A) Boxplot of risk score in age. (B) Boxplot of risk score in gender. (ns, not significant)


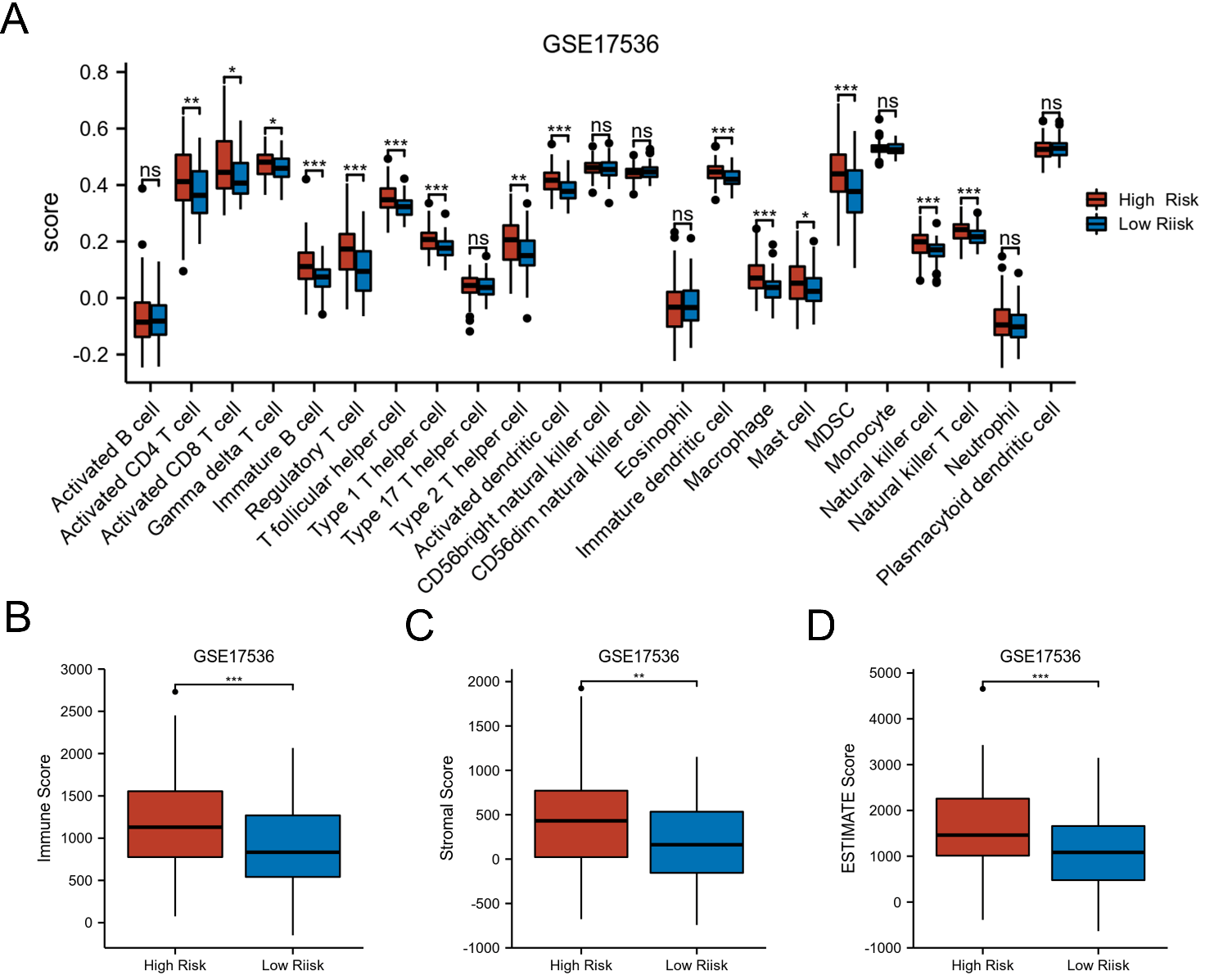


**Supplementary Figure S3.** (A) Infiltration of 23 immune cells using ssGSEA. (B-D) Immune, stromal, and ESTIMATE scores. (*p < 0.05, **p< 0.01, ***p < 0.001, ns, not significant)
